# Supplementary material for: The genome sequence of the hoverfly, Brachypalpoides lentus Meigen, 1822 (Diptera: Syrphidae)
Source: Wellcome Open Res. 2025 Sep 8;10:485. [Version 1] doi: 10.12688/wellcomeopenres.24852.1 (PMC12521903; doi:10.12688/wellcomeopenres.24852.1)
Supplement: Peer review history

# Reviewer report: Reviewer response for version 1

**DOI:** 10.21956/wellcomeopenres.27376.r133662

**Published:** 2025-10-14

**Authors:** Mihajla Djan

**Affiliations:** 1 University of Novi Sad, Novi Sad, Novi Sad, Serbia

The Data Note “The genome sequence of the hoverfly, *Brachypalpoides lentus* Meigen, 1822 (Diptera: Syrphidae)” gives the first genome assembly of the species and it is produced within the Darwin Tree of Life Project. Using Hi-C and PacBio HiFi long-read sequencing, authors generated a chromosome-level assembly comprising two haplotypes. The mitochondrial genome was also assembled. Authors used a wide spectrum of quality assessment tools in order to confirm accuracy of obtained genome sequence. All sequencing data and assembly have been deposited in the European Nucleotide Archive (PRJEB77346) and are openly available. The genome assembled may be used in further genomic studies of the species, as well as in evolutionary genomic research, taxonomy and molecular ecology analyses not only focused on this species, but including research of all hoverflies, as very important pollinator group.

 The rationale for conducting the study is well justified in the Background section. Ecological importance of the species is highlighted, as well as their behavioral characteristics. The justification could be stronger if authors highlighted the potential for comparative genomics with other hoverfly genomes, but it is a minor comment and does not affect the clarity of the rationale. One typo is noticed in part “B. lentus also displaya behaviours”.

 Used protocols are in the alignment with all recommendations and adopted strategies in generating high-quality reference genome. Those include Hi-C, PacBio HiFi long-read sequencing and wide quality control. The assembly showed 6 chromosomes, including X and Y. Methods section provided full information for DNA extraction, library preparation, and assembly workflows, providing study reproducibility.

 The *Brachypalpoides lentus *genome is deposited in the European Nucleotide Archive, Accession number PRJEB77346 and the genome sequence is released openly for reuse. All raw sequence data and the assembly have been deposited in INSDC databases. Assembly metrics and QC plots are clearly presented in Tables and Figures 2–6. The presentation meets open-data standards. Enough information is provided for study replication and for downstream use of generated data.

Are sufficient details of methods and materials provided to allow replication by others?

Yes

Is the rationale for creating the dataset(s) clearly described?

Yes

Are the datasets clearly presented in a useable and accessible format?

Yes

Are the protocols appropriate and is the work technically sound?

Yes

Reviewer Expertise:

population genetics, conservation genetic, molecular taxonomy, phylogenetics

I confirm that I have read this submission and believe that I have an appropriate level of expertise to confirm that it is of an acceptable scientific standard.

# Reviewer report: Reviewer response for version 1

**DOI:** 10.21956/wellcomeopenres.27376.r133660

**Published:** 2025-10-11

**Authors:** Lore Esselens

**Affiliations:** 1 Biology, Royal Museum for Central Africa, Tervuren, Belgium

Mitchell and Cockwood present a clear and well-written manuscript describing the first high-quality genome assembly of *Brachypalpoides lentus*, produced within the Darwin Tree of Life project. While the paper follows the standard DToL genome note format, it represents substantial work and careful attention to quality at every stage. The assembly spans 1 315.37 Mb (95.66%) with haplotype 1 scaffolded into six chromosomes, including the X and Y sex chromosomes, and haplotype 2 assembled to scaffold level. The manuscript provides comprehensive methodological detail, including PacBio HiFi sequencing, Hi-C scaffolding, detailed assembly curation, and multiple quality assessments such as BUSCO and Merqury. The data are clearly presented resulting in a valuable genomic resource to support future research on *Brachypalpoides lentus* and hoverfly biology more broadly. The manuscript would be ready for indexing after only very minor revisions.

 In the Keywords section: *Brachypalpoides lentus* should be in italic.

 In the Background section: “Notably, *B. lentus* also displaya” should be ‘displays’.

 In the Assembly statistics section: the sentence “Table 4 lists the assembly metric benchmarks adapted from Rhie et al. (2021) the Earth BioGenome Project Report on Assembly Standards (September 2024).” seems to miss a connector such as “and” between the two sources. I suggest rephrasing to something like: “Table 4 lists the assembly metric benchmarks adapted from Rhie et al. (2021) and the Earth BioGenome Project Report on Assembly Standards (September 2024).” for clarity.

Are sufficient details of methods and materials provided to allow replication by others?

Yes

Is the rationale for creating the dataset(s) clearly described?

Yes

Are the datasets clearly presented in a useable and accessible format?

Yes

Are the protocols appropriate and is the work technically sound?

Yes

Reviewer Expertise:

Evolutionary genomics

I confirm that I have read this submission and believe that I have an appropriate level of expertise to confirm that it is of an acceptable scientific standard.
